# Somatosensory stimulation treatments for postoperative analgesia of mixed hemorrhoids: Protocol for a systematic review and network meta-analysis

**DOI:** 10.1097/MD.0000000000014441

**Published:** 2019-02-08

**Authors:** An-Mei Zhang, Min Chen, Tai-Chun Tang, Di Qin, Ling Yue, Hui Zheng

**Affiliations:** a The 3rd Teaching Hospital/Acupuncture and Tuina School, Chengdu University of Traditional Chinese Medicine; b Hospital of Chengdu University of Traditional Chinese Medicine, China.

**Keywords:** network meta-analysis, pain after hemorrhoidal surgery, Somatosensory stimulation, study protocol

## Abstract

**Background:**

Pain after hemorrhoidal surgery bothers both clinicians and patients. Somatosensory stimulation treatments have shown promising effect on the pain after hemorrhoidal surgery, but the comparative effectiveness between them has not been studied. We aim to determine the relative effectiveness among these treatments on pain relief after hemorrhoidal surgery by using network meta-analysis.

**Method:**

We will search the following electronic databases: MEDLINE, EMBASE, the Cochrane library, Chinese Biomedicine database (CBM), China National Knowledge Infrastructure (CNKI). We will include randomized controlled trials (RCTs) that examine the effect of somatosensory stimulation treatments on pain after hemorrhoidal surgery. The primary outcome will be the responder rate after treatment. The secondary outcomes will include the assessments with pain intensity scales (visual analog scale, numeric rating scale, or other scales) on day 1 to 7 after surgery. Two independent reviewers will extract needed information from eligible trials using standardized electronic forms. Network meta-analysis will be performed using a frequentist framework based on electrical network theory. The relative effectiveness of the treatments will be ranked by using *P* score, which is the mean probability of a treatment ranking the best in all treatments. Meta-regression will be performed to assess the impact of surgery type, anesthesia methods, and funding source on the treatment ranking. The quality of the eligible RCTs will be evaluated by the Cochrane risk of bias tool.

**Ethics and dissemination:**

The result of this network meta-analysis will clarify which is the relatively best somatosensory-stimulation treatment in relieving postoperative pain caused by hemorrhoidal surgery, and the review will, therefore, guide the management of postoperative pain after hemorrhoidal surgery for clinicians and patients. This review does not require ethical approval and will be reported in a peer-reviewed journal.

**Trial registration number:**

PROSPERO CRD42018115558.

## 1 Introduction

Hemorrhoids are common and frequently-occurring diseases in clinical practice, which seriously affect people's normal life and often manifest as hematochezia, escape, anal pain, heaving pain, discomfort, and so on. Hemorrhoids are cushions of vascular tissue located within the submucosal space and are considered part of the normal anatomy of the anal canal.^[1]^ Some studies have shown that 10 million people in the United States reported hemorrhoids. A peak in prevalence is noted between 45 and 65 years of age;^[2]^ 1 in 3 Americans has hemorrhoids on screening.^[3]^ Hemorrhoids are highly prevalent in general population; 38.9% of the adult population suffered from hemorrhoids, and about 8% of them were classified with grade III or IV hemorrhoids.^[4]^ Among the patients with anorectal diseases, 98% of them had hemorrhoid symptoms. Conservative treatments are first-line treatments for hemorrhoids,^[5,6]^ but surgeries should be performed for patients with grade III or IV hemorrhoids.^[7]^ The evidence of surgical interventions for grade III or IV hemorrhoids are convincing.^[6,8,9]^ The pain after the surgical interventions bothers patients, especially those receiving hemorrhoidectomy.^[10]^ Several treatment options are provided for pain management after hemorrhoid surgery, which includes local suppositories, limited injection of long-acting analgesic drugs, oral administration or intramuscular injection of non-steroidal anti-inflammatory drugs (NSAIDs) or opioid medications.^[11–13]^ These treatments are either with inconclusive evidence^[12,13]^ in their effectiveness or with known adverse events^[11]^ (nausea, vomiting, dizziness, lethargy, urinary retention, etc.); in addition, a recent systematic review shows that unsatisfactory control of postoperative pain is a major problem in Ambulatory hemorrhoidal surgery.^[14]^ These factors urge physicians to find therapeutics with better pain-relief effect and less adverse events.

Somatosensory stimulation treatments are nonpharmacological treatments that apply stimulation (electrical, heat, or manual) to sensory system; they are beneficial for pain management.^[15–18]^ These treatments include acupuncture-related techniques, transcutaneous electrical nerve stimulation (TENS), moxibustion (heat stimulation), and cupping (mechanic stimulation). These treatments have the advantages of lower adverse events, and they were reported to be effective in the management of postoperative pain after hemorrhoidectomy.^[19–21]^ Owing to the large number of somatosensory-stimulation treatments, head-to-head comparisons of all the treatments through randomized controlled trials (RCTs) are time-consuming and financially impossible. Network meta-analysis allows the combination of head-to-head comparisons and indirect comparisons and generates treatment rankings based on currently available evidence. Therefore, we will conduct a network meta-analysis to determine the comparative effectiveness of somatosensory-stimulation treatments in treating postoperative pain after hemorrhoidal surgery.

## 2 Methods

### 2.1 Study design

**Figure 1:**
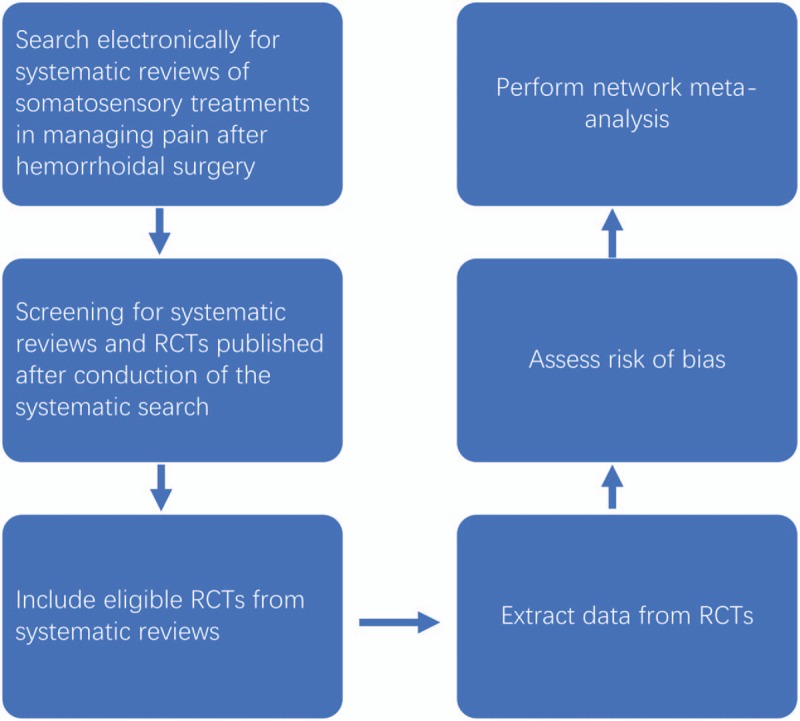
Flow chart of the systematic review and randomised controlled trials.

The study protocol is reported in line with the Preferred Reporting Items for Systematic reviews and Meta-Analyses Protocol (PRISMA-P).^[22]^ The procedure of this review is shown in Figure 1.

### 2.2 Eligibility criteria

#### 2.2.1 Types of studies

We will include RCTs and exclude cohort studies, case series, case reports, reviews, experimental studies focusing on treatment mechanism, in vitro experiments, or animal studies.

#### 2.2.2 Types of participants

We will include trials recruiting patients with postoperative pain following hemorrhoidal surgery. We will not set restrictions on age, gender, region, course of disease, degree of illness, and type of surgery or anesthesia.

#### 2.2.3 Types of interventions

We will include trials examining the effectiveness of somatosensory treatments (hand-acupuncture, electro-acupuncture, TENS, electrical stimulation, acupoint injection, acupoint catgut embedding, acupressure, acupoint application, moxibustion, auricular point sticking) in the management of postoperative pain following hemorrhoidal surgery. These treatments are used alone or in combination. The control group is other somatosensory treatments, usual care, analgesics, NSAIDs, placebo, or waiting-list control. We will exclude trial examining the effectiveness of somatosensory treatments combined with herbal medicine since the effect and its related mechanism of herbal medicine are not clearly elucidated.

#### 2.2.4 Types of outcome measures

The outcomes of eligible RCTs should include pain intensity assessment or responder rate.

### 2.3 Information sources

We will search the electronic databases MEDLINE, EMBASE, the Cochrane Library, Chinese Biomedicine Database (CBM), and China National Knowledge Infrastructure (CNKI) for RCTs examining the effectiveness of the interventions recommended for postoperative analgesia of mixed hemorrhoids. The databases will be searched from inception to May 2018. To ensure that the most recent trials will be listed, we will also search RCTs testing interventions for postoperative analgesia of mixed hemorrhoids in the databases in the year 2018. An experienced librarian will serve to develop a search strategy to find out systematic reviews that examined the effectiveness of the somatosensory stimulations. A combination of terms of medical subject headings (MeSH), keywords will be utilized in the search strategy. The MeSH and keywords contain “hemorrhoid”, “acupuncture analgesia”, “randomized controlled trial”, and synonymous words. If there is any lack of research information, contact the original author to obtain the original information. Language restrictions will not be used in this review. Details of the search strategy are shown in Table 1.

**Table 1:**
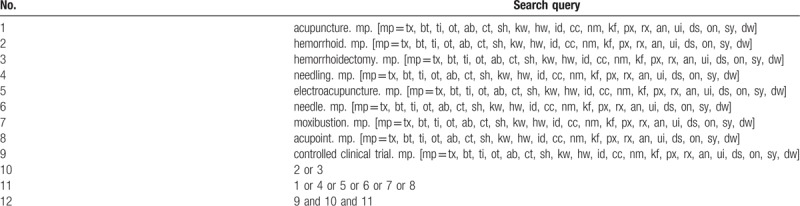
Search strategy.

### 2.4 Study selection

Two independent reviewers selected RCTs according to the eligibility criteria mentioned above, and they determined sample articles to test the data extraction table in pre-extraction period. Any discrepancy in study selection will be solved by team discussion and arbitrated by a third reviewer. Retrieved articles will be imported into Note Express software(version 3. 0) for screening titles and abstracts; full-text articles will be acquired for further screening when the 2 reviewers are unable to decide based on titles and abstracts.

### 2.5 Data extraction

After identification of eligible RCTs, 2 independent reviewers will extract the necessary information from the included RCTs using a standardized form. The form will be developed by a consensus of all the reviewers. Extraction items in the form are shown in Table 2. Characteristics of the RCTs: study, country and publication year; facility; sample size; course of disease; sex ratio; fund support; safety analysis; operation mode; anesthesia mode; disease diagnosis criteria; Characteristics of the interventions of RCTs: the name and method of intervention measures; the number of people in each group; the overall treatment period; main outcome measures: specific figures; collection time point; the number of acupoints; main points; assessment criteria; Risk of bias assessment: whether the random method is correctly generated, whether the random distribution is hidden, whether the blind method is used and whether the use method is correct, whether has the incomplete data and selective reporting, other biases. The numerical data are extracted and calculated to obtain the combined effect quantity. If the data are missing, the researcher will contact the correspondent or the original author to obtain the original data.

**Table 2:**
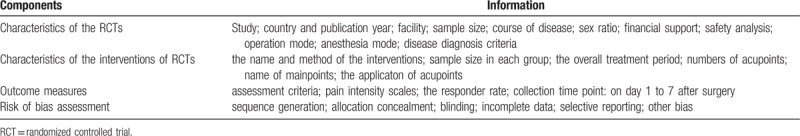
Data extraction.

### 2.6 Outcome assessment

Responder rate will be the primary outcome of this review. A responder is defined as a patient who has at least 30% improvement in pain intensity scales or is being judged as markedly improved according to the following criteria: a visual analog scale (VAS) score ranges from 0 to 3 cm; the average VAS score of the patients on the 3rd or 7th day after the operation is significantly reduced by 4 cm. Secondary outcomes are the pain intensity scales that including VAS, numeric rating scale (NRS), or other scales. According to a previous review of postoperative analgesia of mixed hemorrhoids,^[19,23,24]^ most of the trials report pain assessment at 1 to 7 days after randomization; so we will record data of the outcomes collected during 1 to 7 days after randomization.

### 2.7 Risk of bias assessment

We will choose the Cochrane Collaboration's risk of bias tool^[25]^ to evaluate the methodological quality of RCTs. The risk of bias tool consists of 6 domains: sequence generation, allocation concealment, blinding, incomplete data, selective reporting, and other bias. Two independent reviewers will independently assess the risk of bias of eligible RCTs. Sequence generation will be considered as adequate if central randomization or tables of random numbers are used. Allocation concealment will be considered as tolerable if central randomization or sealed envelopes are used. We will consider blinding as adequate if participants, outcome assessors and statisticians are blinded from the group assignment. Other domains will be assessed exactly as the criteria of the risk of bias tool. A summary of risk of bias of all the 6 domains will be given for each trial. We will consider the sequence generation, allocation concealment and blinding as the essential key domains to score the overall quality of a trial. For each study, all 6 domains will be evaluated and displayed (Table 2). Discrepancies among the 2 reviewers will be solved by discussion or will be assessed by a third reviewer.

### 2.8 Statistical analysis

The network meta-analysis will be conducted using the NetMeta package in the R software (http://www.r-project.org/, version 3. 5. 0). Effect size of a treatment will be presented as standardized mean differences (SMDs) for continuous outcomes (pain intensity scales) or relative ratio (RRs) for categorical outcomes (responder rate), and the 95% confidence intervals (95% CIs) of the SMD and RR will be calculated. Consistency of the network will be evaluated by comparing direct evidence with indirect evidence through *z* test. We will use the comprehensive Cochrane *Q* test to evaluate heterogeneity between and within trials. We will also use a net-heat plot to determine the source of heterogeneity owing to different trial designs. We will use *P* score to describe the mean probability of each intervention being the best measure and to rank the treatments according to the *P* score.

### 2.9 Dealing with missing data

There will be missing data in the trials that we included. We will first contact the authors to ask for original data by email or phone calls, if possible. If the original data are not available, we will try to calculate the data through the available coefficients.

### 2.10 Subgroup analysis

To address the potential heterogeneity and inconsistency across RCTs, we will perform a subgroup analysis. The potential source of heterogeneity may be type of surgery or anesthesia, blinding method (open trial, single-blind, and double-blind), quality of evidence (high risk, unclear of the risk, and low risk), course of disease, and mean age of the participants, main points, number of the points, diagnostic criteria, background therapy, and financial support. Meta-regression models will be used to quantify the difference between subgroups and test for statistical significance.

## 3 Discussion

We will use network meta-analysis to integrate the direct and indirect evidence of somatosensory treatments in the management of postoperative pain and rank the treatments according to the probability and select the best somatosensory treatment and analgesia plan, so as to provide a reference for the application of somatosensory treatments after the operation of clinical mixed hemorrhoids. Clinicians can make the most appropriate choice according to patients, hospitals and their own conditions. It can also enable doctors to improve the treatment methods according to the shortcomings of these methods and conduct further research.

## Acknowledgments

The authors would like to thank Tao-Hong He in colorectal department of the Teaching Hospital of Chengdu University of Traditional Chinese Medicine for advice in the treatment of postoperative pain.

## Author contributions

Contributors: AMZ, MC, TCT, DQ, and HZ contributed to the conception and design of the study protocol. The search strategy was developed and run by AMZ and HZ, who will also screen the title and abstract of the studies after running the search strategy. HZ and MC will also screen full copies of the remaining studies after title and abstract selection, while MC and TCT will extract information of the identified studies; MC will check the data entry for accuracy and completeness. DQ and HZ will give advice for data analysis and presentation. All the authors drafted and revised this study protocol and approved it for publication.

**Conceptualization:** An-Mei Zhang, Hui Zheng.

**Writing – original draft:** An-Mei Zhang.

**Writing – review & editing:** An-Mei Zhang, Min Chen, Tai-Chun Tang, Di Qin, Ling Yue, Hui Zheng.

**Data curation:** Min Chen.

**Funding acquisition:** Min Chen, Hui Zheng.

**Methodology:** Min Chen, Tai-Chun Tang, Ling Yue.

**Formal analysis:** Di Qin, Hui Zheng.

Hui Zheng orcid: 0000-0002-0494-1217.
